# Advances in the study of cholinergic circuits in the central nervous system

**DOI:** 10.1002/acn3.51920

**Published:** 2023-10-16

**Authors:** Ganghua He, Yang Li, Hua Deng, Hongyan Zuo

**Affiliations:** ^1^ Beijing Institute of Radiation Medicine Beijing China; ^2^ College of Life Science and Engineering, Foshan University Foshan China

## Abstract

**Objective:**

Further understanding of the function and regulatory mechanism of cholinergic neural circuits and related neurodegenerative diseases.

**Methods:**

This review summarized the research progress of the central cholinergic nervous system, especially for the cholinergic circuit of the medial septal nucleus‐hippocampus, vertical branch of diagonal band‐hippocampus, basal nucleus of Meynert‐cerebral cortex cholinergic loop, amygdala, pedunculopontine nucleus, and striatum‐related cholinergic loops.

**Results:**

The extensive and complex fiber projection of cholinergic neurons form the cholinergic neural circuits, which regulate several nuclei in the brain through neurotransmission and participate in learning and memory, attention, emotion, movement, etc. The loss of cholinergic neurotransmitters, the reduction, loss, and degeneration of cholinergic neurons or abnormal theta oscillations and cholinergic neural circuits can induce cognitive disorders such as AD, PD, PDD, and DLB.

**Interpretation:**

The projection and function of cholinergic fibers in some nuclei and the precise regulatory mechanisms of cholinergic neural circuits in the brain remain unclear. Further investigation of cholinergic fiber projections in various brain regions and the underlying mechanisms of the neural circuits are expected to open up new avenues for the prevention and treatment of senile neurodegenerative diseases.

## Introduction

The central cholinergic nervous system is the main neurotransmitter system in the brain and is composed of cholinergic neurons that can synthesize acetylcholine (ACh). Acetylcholine transferase (ChAT) is a marker enzyme for cholinergic neurons, and acetylcholinesterase (AChE) is synthesized in cholinergic neurons. Cholinergic neurons are mainly distributed in the cerebral cortex, hippocampus, and basal forebrain. Downstream neurons projected by cholinergic fibers possess nicotinic acetylcholine receptors (nAChRs) and muscarinic acetylcholine receptors (mAChRs). Neurons at all levels are connected by synapses to form extensive and complex neural loops that participate in learning and memory, emotion, speech, and other skills.

The cholinergic neurons in the brain can be divided into two types: local loop and projective. Local loop neurons are intermediate neurons that form a loop within the nucleus and do not project outside it. Local loop neurons are mainly located in the striatum, nucleus accumbens, olfactory tubercle, hippocampus, and cerebral cortex layers II–V. The majority of cholinergic projection neurons are from the basal forebrain and brainstem to other brain regions, which constitute the basal forebrain and brainstem cholinergic system, respectively.

Since the 1980s, the distribution and fiber projection of cholinergic neurons in the basal forebrain has attracted much attention.^1^, ^2^, ^3^ Cholinergic neurons in the basal forebrain and brainstem were divided into the Ch1–Ch6 group by Mesulam in 1983, in which Ch1–Ch4 are located in the basal forebrain and for basal forebrain complex. Moreover, Ch1, Ch2, and Ch3 were named the medial septal nucleus (MS), vertical part of diagonal band (VDB), and horizontal part of diagonal band (HDB), respectively, Ch4 contained some areas with indistinct boundaries, such as the preoptic large cell area, an anonymous substance of the globus pallidus floor, the extending forward ventral globus pallidus (substriatal gray matter), and the basal nucleus of Meynert (NBM).^4^ These structures contain different types of cholinergic neurons. Although they have different transmitter content, morphology, and projection patterns, together they provide highly extensive, multi‐branched cholinergic inputs to the entire cortical mantle, hippocampus, thalamus, and amygdala^1^ and receive synaptic inputs from the ventral and dorsal striatum, hypothalamus, amygdala, and brainstem tegmentum. With the signal transduction mediated by the efferent and afferent fibers of cholinergic neurons, the basal forebrain complex is projected to the cortex and hippocampus through the cingulate gyrus around the corpus callosum or through the screen nucleus, external capsule, and uncinate tract to form a neural loop and participate in the regulation of mood, attention, spatial memory, and cognition.

The brainstem cholinergic system consists of pontine tegmental cholinergic neurons and medulla oblongata cholinergic neurons. The pontine tegmental cholinergic system is mainly composed of cholinergic neurons in the interpeduncular tegmental area of the Ch5 group and the dorsolateral or lateral tegmental nucleus of the Ch6 group. The fibers are divided into the dorsal tegmental tract and ventral tegmental tract, which project to the thalamus, hypothalamus, globus pallidus, caudate putamen, and form a reticular ascending activation system together with other ascending fibers to participate in functional regulation, such as awakening and alertness of the body. Cholinergic neurons in the medulla oblongata are mainly located in the medial area of the facial nucleus of the ventral respiratory center of the medulla oblongata. nAChRs are distributed on the cell membrane of respiratory central neurons, which play a regulatory role in the function of the respiratory center of the medulla oblongata. In addition, the cell bodies and visceral motor fibers of cholinergic neurons in the cranial nucleus constitute the cranial nerve and participate in the regulation of somatic and visceral movement.

Recent studies have shown that the central cholinergic nervous system, especially the highly complex basal forebrain cholinergic system, is not only related to cortical activation, emotion, attention, sensory coding, motivation, and memory but it is also involved in neural diseases, such as Alzheimer's disease (AD), Parkinson's disease (PD), schizophrenia, autism, attention deficit disorder, and drug abuse.^5^, ^6^, ^7^ The cholinergic neuronal network and its morphology in the basal forebrain are complex, and the surrounding noncholinergic neurons are heterogeneous, resulting in diverse signaling pathways. Therefore, further understanding of the function and regulatory mechanisms of central cholinergic circuits is important for the study of brain function and the prevention and treatment of neurodegenerative diseases.

## Medial Septal Nucleus‐Hippocampal Cholinergic Loop

**Figure 1:**
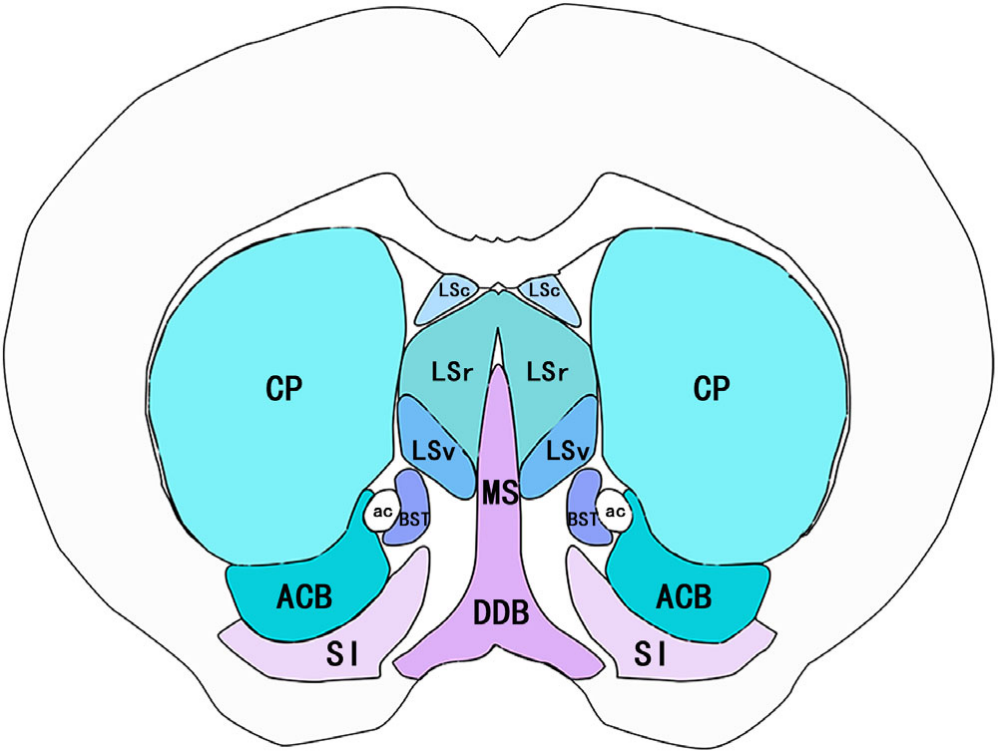
Distribution of the medial septal nucleus, diagonal band of Broca, and the surrounding nuclei. MS, medial septal nucleus; DDB, diagonal band of Broca; BST, bed nucleus of the stria terminalis; ac, anterior commissure; LSv, lateral septal nucleus, ventral part; LSr, lateral septal nucleus, rostral (rostroventral) part; LSc, lateral septal nucleus, caudal (caudodorsal) part; CP, caudoputamen; ACB, nucleus accumbens; SI, substantia innominate.

The septal nucleus is the subcortical nucleus of the septum. The septum includes the structures under the corpus callosum and its surrounding area and is located in the medial wall of the cerebral hemisphere above the endplate and anterior commissure. According to spatialization,^8^ the septal nucleus can be divided into four subnuclear groups: dorsomedial nucleus (medial septal nucleus and diagonal band nucleus), ventrolateral nucleus (lateral septal nucleus), dorsal nucleus (bed nucleus of the stria terminalis), and caudal nucleus (hippocampal fimbria nucleus and triangular septal nucleus), which we illustrated in Figure 1. The medial septal nucleus is a dorsomedial nucleus located in the deep septal nucleus of the septal region. It has a complex fiber connection with the surrounding structures, and the connection with the hippocampal formation is established by the fiber projection of the septal‐hippocampal pathway. Broca et al.^9^ speculated that most fibers in the septal area were linked to the posterior part of the corpus callosum and established a direct relationship with the hippocampus. The hippocampus, which is located between the thalamus and the medial temporal lobe, is a part of the limbic system. As a high‐level neural center of learning and memory, the hippocampus plays a critical role in the storage and conversion of memory and orientation.

**Figure 2:**
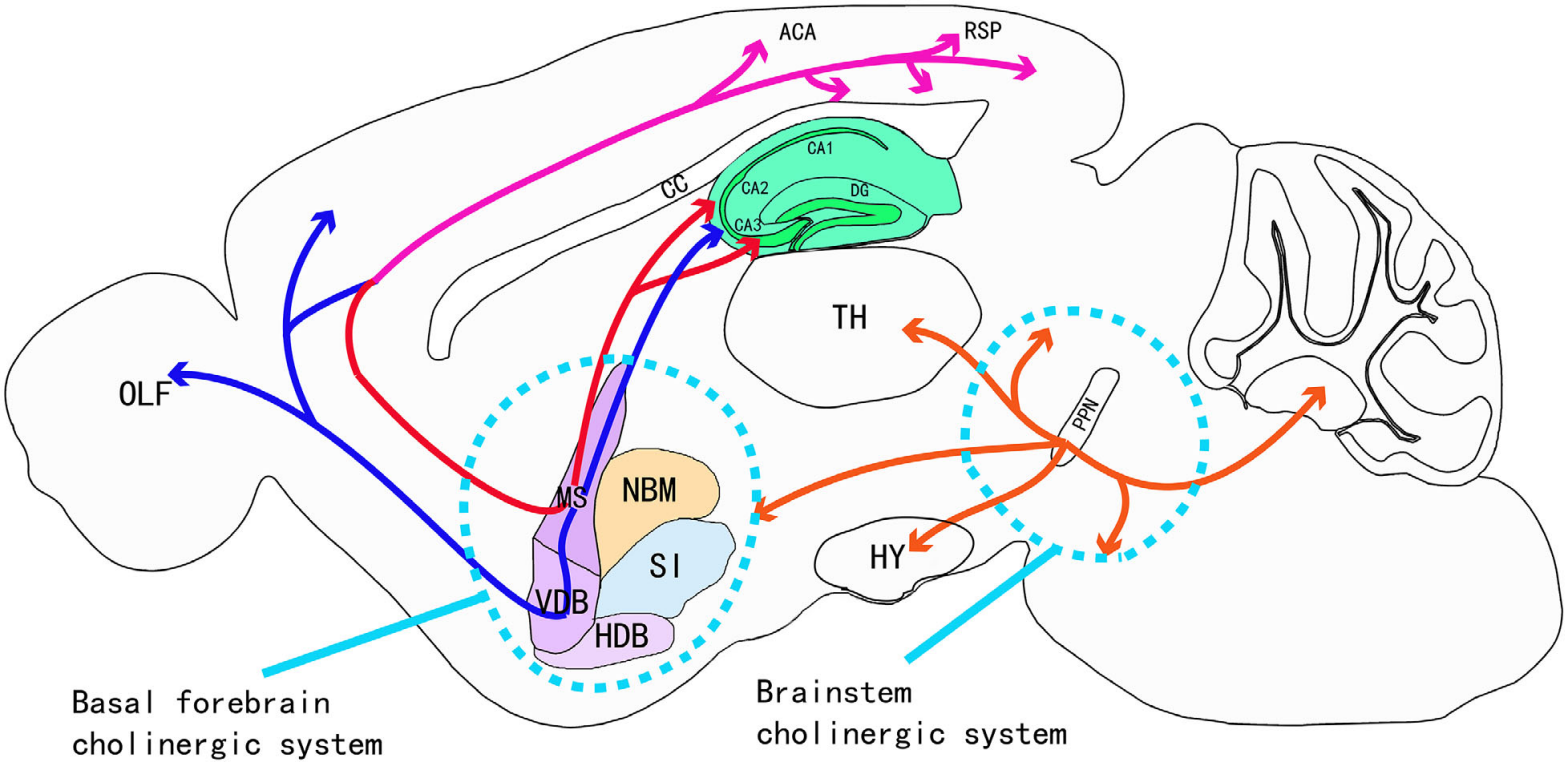
Cholinergic circuits in the brain. Cholinergic neurons in the medial septal nucleus project through the fimbria, dorsal fornix, and amygdaloid complex to homocortical targets in the hippocampus (red curve), as well as the entorhinal area, cingulate area, retrosplenic area, and hypothalamic cortex (pink curve). The DDB nerve fibers project to the fornix and fimbria via the MS. The projection fibers reach the hippocampus through the amygdala, and the nerve fibers also project to the olfactory bulb and cerebral cortex (blue curve). PPN cholinergic cell bodies are widely projected to the thalamus, tectum, substantia nigra, basal forebrain, basal ganglia, reticular formation, raphe nucleus, locus ceruleus, cerebral nucleus, pons, and deep cerebellar nucleus (orange curve).

**Figure 3:**
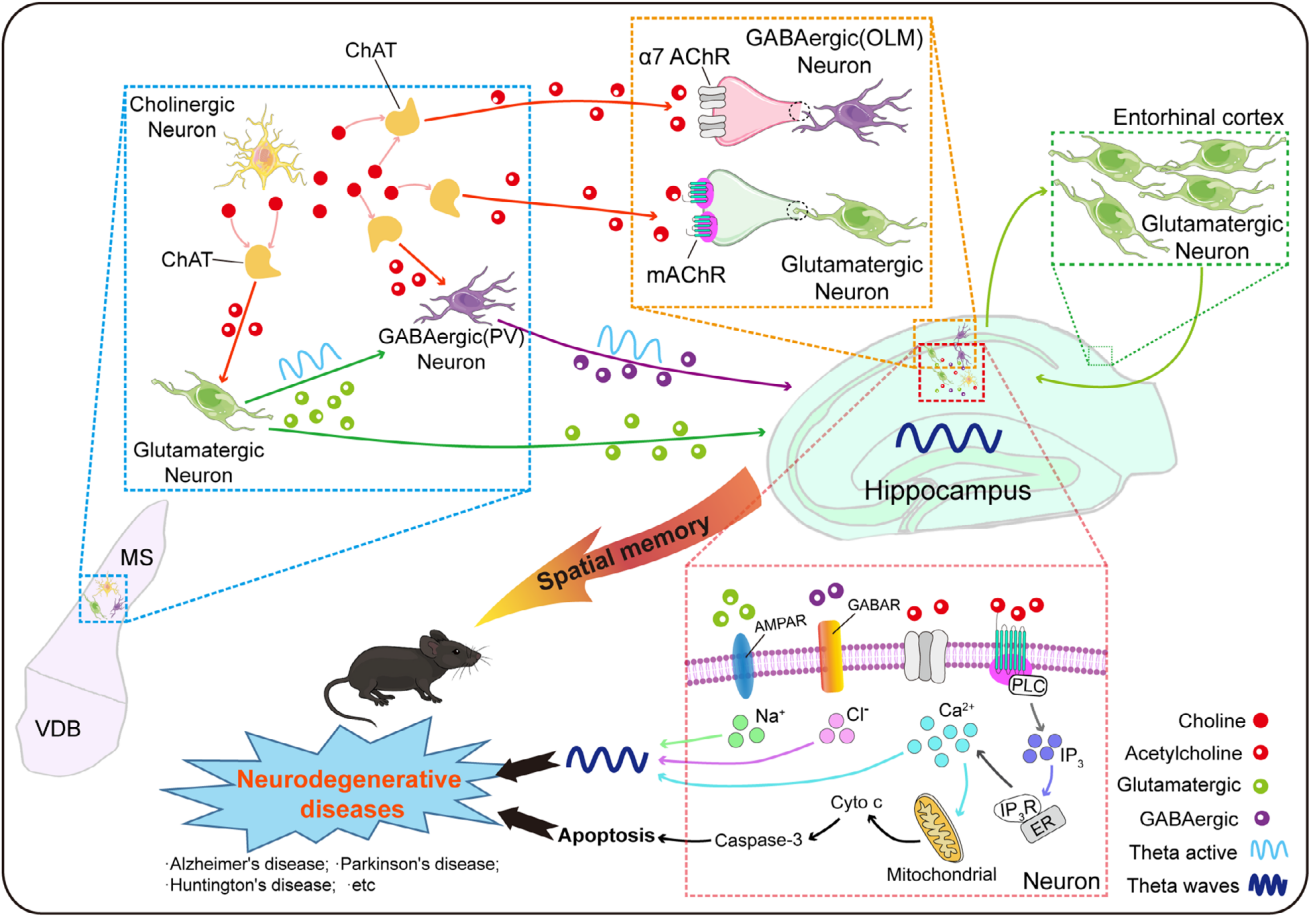
Medial septum cholinergic neurons mediated theta oscillations in the hippocampus. The activation of cholinergic neurons in the medial septum can modulate theta oscillations in the hippocampus through two pathways. Firstly, cholinergic projections from the medial septum to the hippocampus directly recruit different subgroups of neurons via distinct receptor subtypes, regulating theta oscillations. Secondly, cholinergic neurons in the medial septum regulate hippocampal circuitry and theta expression through interactions with glutamatergic and GABAergic neurons. Cholinergic transmission in the medial septum activates adjacent glutamatergic and GABAergic neurons, thereby inducing theta oscillations in the hippocampus. Both abnormal theta oscillations and neuronal apoptosis of hippocampus are involved in the deficiency of spatial memory. The underlying molecular mechanisms may include neurotransmitter‐mediated changes of intracellular ions and the mitochondria‐dependent Cyto c – caspase 3 pathways, ultimately resulting in neurodegenerative diseases. Cyto c, cytochrome c; ER, endoplasmic reticulum; PLC, phospholipase C; IP_3_, inositol 1, 4, 5‐trisphosphate; IP_3_R, inositol 1, 4, 5‐trisphosphate receptor.

MS cholinergic neurons project from the medial septal nucleus and diagonal band nucleus through the hippocampal fimbria, dorsal fornix, and amygdaloid complex to the homotopic cortical targets in the hippocampus, as well as the entorhinal, cingulate, postsplenic, and hypothalamic cortices,^10^, ^11^ providing cholinergic inputs to the hippocampus and entorhinal cortex (Fig. 2). It has been reported that in the Ch1 nuclei of the basal forebrain of primates, MS cholinergic neurons located in the diagonal zone form a continuous, vertically oriented column. Although only about 10% of MS cholinergic neurons project to the hippocampus, these projections are the main source of cholinergic nerves in the hippocampus and play an important role in regulating its excitability.^1^, ^12^, ^13^, ^14^ Recent studies have shown that stimulating cholinergic neurons in the MS can regulate the hippocampus directly through the basal forebrain‐hippocampal cholinergic projection. Cholinergic transmission in the MS projects to the hippocampus and recruits different subpopulations of neurons through various receptor subtypes to regulate theta oscillations.^15^, ^16^, ^17^ Another pathway from the MS to the hippocampus is found via MS cholinergic signals that first project to the internal noncholinergic neurons and then indirectly regulate the hippocampus by noncholinergic neuron projection.^18^ The mechanisms include activation of neighboring glutamatergic and GABAergic neurons by cholinergic transmission in the MS, subsequently inducing theta oscillations in the hippocampal region.^16^, ^17^ The two pathways from the MS to the hippocampus work together to maximize the synchronization of hippocampal discharges and theta oscillations.^12^, ^19^ The underlying molecular mechanisms may include neurotransmitter‐mediated changes of intracellular ions, and the following depolarization of cell membrane, ultimately affecting theta oscillations in hippocampus. On the other hand, the mitochondria‐dependent Cyto c‐caspase 3 pathways may be activated by intracellular calcium changes, leading to neuronal apoptosis. Both abnormal theta oscillations and neuronal apoptosis have been reported to be involved in neurodegenerative diseases.^20^, ^21^ Altogether, MS cholinergic neurons mediate hippocampal theta oscillations through two pathways, which are involved in neurodegenerative diseases (Fig. 3).

The MS is a pacemaker of the hippocampal theta rhythm.^22^ Cholinergic and γ‐aminobutyric acid (GABA) neurons in the MS regulate the excitability of hippocampal neurons through the release of neurotransmitters, thereby affecting the learning ability and spatial memory of animals. Hippocampal theta oscillations are thought to represent a balance between memory coding and retrieval.^23^, ^24^ By regulation of AMPA and NMDA receptors, mAChRs mediated activation of PV interneurons and affected high‐frequency oscillatory activity and synchronization in the hippocampus.^25^ At the brain network level, the balance between gamma and theta oscillations in the hippocampus has been proven to play an important role in regulating learning and memory.^17^ In the cholinergic projection pathway, theta‐gamma coupling is associated with spatial working memory processes, and the reduced theta‐gamma coupling is linked to cognitive impairments in aging and AD.^16^, ^26^ mAChR activation may induce multiple overlapping oscillation modes, such as theta and alpha, beta, and gamma oscillations. The agonists of mAChR or mGluR can produce theta or gamma oscillations,^27^ respectively, and improve memory.^12^, ^25^, ^28^, ^29^, ^30^ The results of microdialysis experiments^31^, ^32^ showed that in the process of memory activity, the level of ACh in the hippocampus increased, and the expression of nAChRs in the hippocampus was upregulated,^33^ while blocking the local mAChR signal in the hippocampus damaged memory.^34^ Reportedly, cholinergic nerves projected from the basal forebrain to the hippocampus can activate the theta rhythm in the hippocampus, which plays an antiepileptic role.^35^, ^36^, ^37^ In summary, cholinergic projections from the MS to the hippocampus, through ACh and its signal transduction, affect neuronal excitability and theta oscillations in the hippocampus, which is important for the regulation of spatial learning and memory, correlated with the progression of neurodegenerative diseases including AD and PD, as summarized in Table 1.

**Table 1:** Major cholinergic projections in the brain and its related neurodegenerative disease.

Type	Origin	Nucleus group	Main projections	Functions	Related diseases	References
The basal forebrain cholinergic system	MS	Ch1	MS → Hippocampus, MS → Olfactory area, MS → Cingulate area, MS → Splenic posterior area, MS → Hypothalamic cortex	Release of ACh, activation of Theta oscillation in the hippocampus, regulation of learning and spatial memory	Epilepsy, aging, AD, PD	[10, 16, 17, 23, 24, 26, 35, 36, 37]
	DBB(VDB)	Ch2	VDB → MS → Hippocampus, VDB → Amygdala → Hippocampus	ACh induces hippocampal Theta rhythm through MS under the action of ChAT, regulating cognitive function	DLB，PDD, PD	[4, 38, 39, 40, 41]
	DBB(HDB)	Ch3	HDB → olfactory bulb, HDB → S1 cortical regions, HDB → A1 cortical regions, HDB → V1 cortical regions, HDB → mPFC	Regulation of the olfactory system. Associated with specific sensory processing required for attention or learning processes	Olfactory dysfunction associated with PD, mild cognitive impairment, and AD	[42, 43, 44]
	NBM	Ch4	NBM → Frontal lobe, NBM → Cingulate cortex, NBM → Temporal pole, NBM → Parietal, NBM → occipital cortices	Associated with executive dysfunction, memory impairment, spatial dysfunction related to vision, and hallucinations	AD, PDD, DLB, progressive supranuclear palsy, corticobasal syndrome, alcoholic dementia	[45, 46, 47, 48, 49, 50]
The brainstem cholinergic system	PPN	Ch5	PPN → Thalamus, PPN → Basal forebrain, PPN → Basal ganglia, PPN → Reticular formation, PPN → Locus coeruleus, PPN → Cranial nerve nuclei. PPN → Dorsolateral striatum	Controls the flexibility of motor actions, gait, and posture	Involved in the occurrence of gait freezing in PD. Associated with DLB and PDD	[10, 51, 52, 53, 54, 55]
	LDT	Ch6	LDT → Thalamus, LDT → Dorsomedial striatum, LDT → Nucleus accumbens	Regulating sensory movement, cognition, and emotions	PD, progressive supranuclear palsy, and dystonia	[40, 56, 57, 58, 59, 60, 61]

## Vertical Branch of Diagonal Band‐Hippocampal Cholinergic Loop

The diagonal band of broca (DBB) is closely connected to the MS in terms of its structural organization. Besides the MS‐hippocampal cholinergic pathway, the connection between the vertical limb of the diagonal band and the hippocampus is another important cholinergic pathway. DBB is located on the dorsal and medial sides of the septum accumbens, alternating with the midline pellucid septum. At the back, the forelimb of anterior commissure protrudes, and DBB splits into a more dispersed structure, with interspersed fibers of gray matter stripes. Outside the parolfactory region, the direction of the white matter fibers in the DBB becomes more diagonal.^62^ The anterior commissural chiasma of the DBB terminates in the medial ventral striatum parallel to the bottom of the forebrain.^9^ The cholinergic cells projecting from the DBB to the hippocampus are located in the Ch2 nucleus group, which is called the VDB. The VDB nerve fibers project to the fornix and fimbria via the MS, and the projection fibers reach the hippocampus through the amygdala.^4^, ^38^ Only 10% of the cholinergic neurons in the MS serve as the main source of cholinergic inputs to the hippocampus. However, cholinergic neurons in the VDB project to hippocampus through the MS, and most of the cholinergic inputs to the hippocampus may come from the VDB. The Ch3 nucleus group is a neuron band near the ventral side of the commissural area, which projects to the olfactory bulb, and it serves as an important source of cholinergic input that regulates the olfactory system,^42^ also known as HDB.^63^ In primate brains, most (70%) of VDB neurons express ChAT, which is much higher than HDB (1%).^1^

Similar to the HDB, the CA2 subregion of the hippocampus is a relatively unexplored region in the human brain. A connection between MS‐VDB and CA2 has been identified in the mouse brain.^64^ Lewy neurites were found confined to the CA2 subregion of the hippocampus in Lewy body dementia (DLB),^39^, ^40^ and Lewy pathological changes were obvious only in the hippocampal CA2 subregion of patients with DLB and in patients with PD. Neurodegeneration of cholinergic fibers is obvious in patients with PD with mild cognitive impairment, which is significantly correlated with the loss of cholinergic neurons in the VDB.^41^ Atrophy and loss of cells in the VDB occur in some neurological diseases.^40^, ^65^, ^66^, ^67^ The accumulation of specific proteins and the loss of cholinergic neurons in the hippocampus of patients with DLB is thought to be associated with the loss of cholinergic neurons in the VDB. Moreover, the loss of cholinergic neurons in the MS Ch1 and Ch2 groups was obvious in patients with DLB.^66^ A significant loss of ChAT‐positive neurons in the VDB was observed in patients with Lewy somatic disorder with cognitive impairment, but the loss of ChAT‐positive neurons in the VDB was not observed in patients with AD and PD without cognitive impairment.^68^ In addition, Hall et al.^40^ found that the neurodegeneration of cholinergic neurons in the hippocampus of patients with Parkinson's disease dementia (PDD) was significant compared with that of the PD and control groups. Altogether, the degeneration and loss of cholinergic neurons in the VDB may lead to an imbalance of the VDB‐hippocampus neural loop, a decrease in ACh, abnormal synthesis and metabolism of ACh, a loss of cholinergic neurons, resulting in abnormal theta oscillations of the hippocampus, ultimately leading to cognitive impairment and an increase risk of neurological diseases.

In summary, the VDB projects cholinergic fibers to the hippocampal region through the MS and induces theta rhythms in the hippocampus, participating in the regulation of cognitive functions. Further investigations of the cholinergic loop projected from the horizontal and vertical branches of the diagonal band to the hippocampus is necessary, especially for understanding and treating cognitive disorders, such as DLB, PD, and AD.

## Meynert Basal Nucleus‐Cortical Cholinergic Loop

In addition to the MS/VDB‐hippocampal cholinergic circuit regulating theta oscillations related to neurodegenerative diseases, the basal forebrain‐cerebral cortex cholinergic circuit also involved in functions related to the Meynert basal nucleus (NBM). Chaves et al.^43^ demonstrated that the HDB/VDB region is primarily associated with the primary sensory cortex and exhibits specific projections to the primary somatosensory (S1), auditory (A1), and visual (V1) cortical regions based on the distribution of its neck and tail neurons. Furthermore, it has bidirectional projections to the prefrontal cortex's anterior/ventral regions (PL/IL) in the medial prefrontal cortex (mPFC), which are associated with olfactory dysfunction in neurodegenerative diseases such as PD and AD.^44^ The NBM projects to most sensory cortex regions, including frontal sensorimotor, parietal, temporal, cingulate gyrus, and visual cortex,^69^ with a wider range of targets in the sensorimotor cortex, but without a specific pattern. Therefore, the correlation of the Meynert basal ganglion‐cortical cholinergic circuit with neurodegenerative diseases is of great significance.

NBM is the main cholinergic nucleus in the Ch4 nucleus group of the basal forebrain, which is located on the anonymous substance and ventral side of the basal forebrain. Previous anatomical studies have shown that the NBM extends from the front of the olfactory tubercle to the uncinate hippocampus, with a length of 13–14 mm in the sagittal plane, and provides most of the cholinergic innervation to the cerebral cortex. According to the characteristics of morphology and connectivity, the anterior, middle, and posterior NBM can be divided into four subregions: the anterior NBM, including the anteromedial (Ch4am) and anterolateral (Ch4a1) subregions; the middle NBM including the Ch4a1 and intermediate (Ch4i) subregions; and the posterior NBM, including the Ch4i and posterior (Ch4p) subregions.^45^

**Figure 4:**
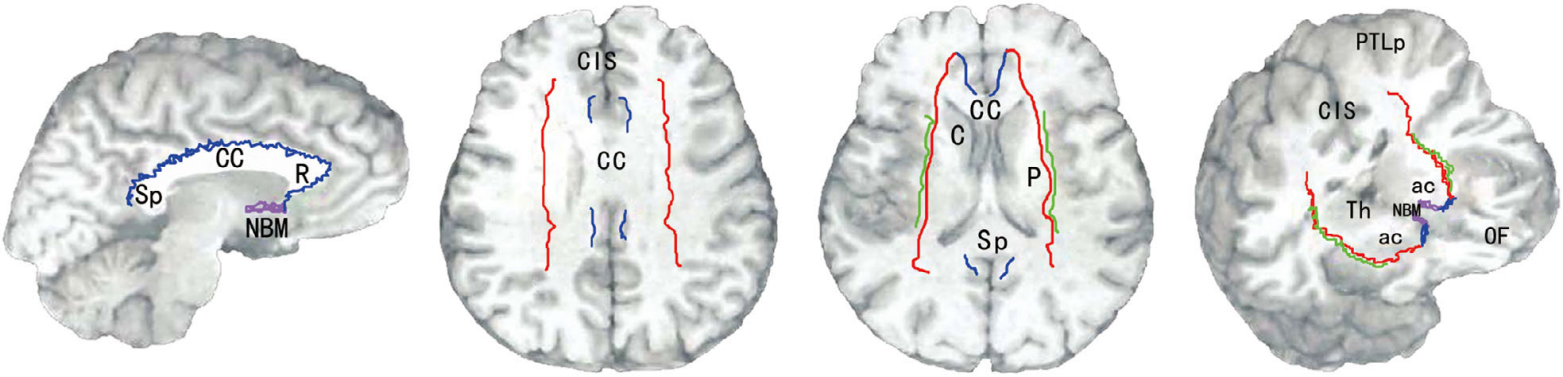
Cholinergic pathway originating from the basal nucleus of Meynert. The medial pathway runs within the cingulate, supplying the anterior orbital lobe, subcingulate, cingulate gyrus, and retrosplenic cortex (blue curve). The lateral capsule pathway runs in the external capsule and uncinate tract, supplying the putamen, amygdala, middle and inferior gyrus, parahippocampal gyrus, and dorsal frontal parietal neocortex (red curve). The lateral pathway of perithecal membrane components runs in the shielded area, supplying the insular, superior temporal gyrus and ventral frontal parietal lobe neocortex (green curve). R, rostrum of corpus callosum; CC, corpus callosum; Sp, splenium of corpus callosum; Cis, cingulate sulcus; C, caudate; P, putamen; OF, orbitofrontal cortex; Th, thalamus; PTLp, posterior parietal association areas. Modified from Selden, N.R., Gitelman, D.R., Mesulam, M.M., 1998. Trajectories of corticopetal cholinergic pathways within the cerebral hemispheres of the human brain. NeuroImage, 7, S26.

Selden et al.^46^ used a histochemical method to trace the white matter fibers originating from NBM with high‐level AChE and identified two main pathways originating from Ch4. The medial pathway runs in the cingulate, supplying the anterior orbital lobe, subcingulate gyrus, cingulate gyrus, and retro‐splenic cortex. The lateral pathway can be subdivided into capsule and perithecal membrane pathways. The capsule pathway runs in the outer capsule and uncinate tract, supplying the putamen, amygdala, middle and inferior gyri, parahippocampal gyrus, and dorsal frontal parietal neocortex. The perithecal membrane pathway travels to the shielding region, supplying the insular, superior temporal gyrus, and ventral frontal parietal lobe neocortex. The dorsal prefrontal cortex receives projections from medial NB/SI neurons, whereas the ventral areas of the prefrontal cortex receive projections from the lateral cholinergic neurons in the basal forebrain.^70^ Most of the lateral parts of the cortex and subcortex receive projections from lateral and posterior cholinergic neurons.^38^ Liu et al.^45^ found that the projection from the anterior NBM mainly dominates the frontal lobe and cingulate cortex, which may be related to executive dysfunction, whereas the projection from the posterior NBM mainly dominates the temporal pole, which may be related to memory impairment. The middle NBM projects to the parietal and occipital cortices and may be involved in vision‐related spatial dysfunction and hallucinations (Fig. 4).

More than 90% of large cell neurons in the NBM are cholinergic neurons. Studies have shown that the loss of cholinergic markers in the cerebral cortex and a decrease in the number of neurons in the basal forebrain^71^ are characteristics of AD, and the degeneration of the basal forebrain system is correlated with early cognitive impairment in AD.^47^, ^48^, ^49^ In PD, the loss of neurons in the NBM is as high as 80%, which is even more serious in the brains of patients with PDD.^72^, ^73^ Neuronal loss in the NBM has been reported to be closely related to cortical cholinergic deficiency and cognitive impairment in AD and PDD.^72^, ^74^ The loss of neurons in the NBM reduces the level of acetylcholine in the cortex, resulting in dysfunction of the cholinergic system and degeneration of the NBM, ultimately leading to cognitive‐related pathological damage and the occurrence of neurodegenerative diseases such as PD and AD.^50^ Several studies have demonstrated the regulation of deep brain electrical stimulation (DBS) in cholinergic neurons. Low‐frequency electrical stimulation of the NBM can promote the release of ACh by cholinergic neurons in the basal forebrain, thus alleviating and improving cognitive impairment in patients with PDD and AD.^75^, ^76^

Overall, the NBM is one of the most important cholinergic nuclei in the basal forebrain, playing a critical role in behavior, memory, attention, vision, and hallucination. Clinically, the DBS of NBM has been used to increase cholinergic fiber projection in the basal forebrain for the treatment of dementia. The method of using DBS to stimulate NBM through transmitting bioelectric impulses to restore distal cholinergic fiber projection can make up for the lack of cholinergic input from the NBM to the cortex, which is promising for the treatment of neurodegenerative diseases such as PDD and AD.

## Other Cholinergic Pathways in the Brain

### Amygdala cholinergic loop related to emotional memory

As a part of the limbic system, the amygdala is a collection of nuclei located in the dorsomedial prefrontal lobe below the central cortex, top forward of the hippocampus, and the lower horn of the lateral ventricle. Three subregions are observed in neuroimaging of the human amygdala: dorsal, ventrolateral, and medial.^77^ The dorsal region consists of the central (CEA) and medial (MeA) nuclei, and the ventrolateral region refers to the basolateral complex (BLA), which includes the lateral nucleus, basolateral nucleus, basomedial nucleus, and basoventral nucleus.^78^ The amygdala plays a pivotal role in the cortex, striatum, and subcortex,^79^ and its afferent fibers originate from NBM cholinergic neurons in the basal forebrain, olfactory bulb, preolfactory nucleus, ventromedial hypothalamic nucleus, and other nuclei. Through the stria terminalis pathway (from the medial cortical nucleus to the nucleus of the stria terminalis, hypothalamus, preoptic area, and septal nucleus) and ventral amygdala (from the basolateral nucleus, inward to the medial part of the bed nucleus of the terminalis, forward to the preoptic area, ventromedial hypothalamic nucleus, dorsomedial thalamic nucleus, and finally to the prefrontal cortex), the efferent fiber projection of the amygdala forms a two‐way interconnection with the medial frontal lobe, septal area, hippocampus, thalamus, striatum, temporal cortex, motor cortex, etc., and thus participates in the regulation of emotion, learning, and memory.^8^ Unlike hippocampus‐dependent spatial memory, emotional memory is largely mediated by the amygdala. The amygdala receives dense NBM cholinergic projections and promotes memory formation.^64^, ^79^ Cholinergic signals are essential for encoding emotional memory.^80^ For example, short‐term and high‐frequency stimulation of the presynaptic fibers of the amygdala participates in the formation of emotional memory mediated by acetylcholine, and emotional memory is strengthened by cholinergic stimulation in the amygdala.^81^

### Pedunculopontine nucleus cholinergic circuits associated with behavioral flexibility

The main motor regulatory area of the brainstem is located in the pedunculopontine tegmental nucleus (PPN), also known as the pedunculopontine nucleus (Ch5 group). PPN is situated in the rostral brainstem and is involved in motor circuits, primarily targeting the dorsolateral striatum.^56^, ^57^ This nucleus plays a role in the control of action, gait, and postural flexibility.^82^ As the dorsal nucleus of the medial pontine thalamic system, the PPN mainly contains cholinergic neurons, γ‐aminobutyric acid neurons, glutamatergic neurons, and glycine neurons.^83^ The cholinergic cell bodies of the PPN are projected to many targets in the thalamus, tectum, substantia nigra, basal forebrain, basal ganglia, reticular formation, raphe nucleus, locus ceruleus, cerebral nucleus, pons, and deep cerebellar nucleus.^10^, ^51^, ^52^, ^53^, ^54^ Retro‐tracing results showed the double‐labeling cholinergic cells of PPN distributed in several brain regions, further indicating the multi‐projection of PPN neurons. It has been proven that the PPN can regulate behavioral flexibility by reward, especially by increasing the sensitivity of the positive reward.^84^, ^85^, ^86^ Some studies have also shown that PPN cholinergic neurons participate in the regulation of gait disturbance and postural instability in patients with PD, with a decrease in dopaminergic neurons in the substantia nigra and the striatum.^15^ The decrease and loss of cholinergic fiber projection were observed in the early stage of PD, and the degeneration and loss of cholinergic neurons in the PPN were positively correlated with the development of PD.^87^ In patients with PD, the PPN is involved in the occurrence of frozen gait,^55^ and it is difficult for patients with frozen gait symptoms to adjust their behavior with any flexibility, such as adjusting the gait speed by changing the gait frequency.^88^ In summary, as an important member of the brainstem cholinergic loop, the PPN can transmit neural signals from the cerebral cortex to the thalamus mediated by cholinergic neurons, thus participating in the cortical excitation and behavior control of the thalamus, and indirectly participating in the regulation of motor function. Moreover, the PPN‐mediated cholinergic loop might play an important role in the occurrence and development of PD.

### The laterodorsal tegmental nuclei and the local cholinergic circuits in the striatum

The cholinergic projections in brainstem originate from both PPN and laterodorsal tegmental nuclei (LDT), which together innervate the lateral hypothalamic area. The LDT, located in the caudal brainstem (Ch6 group), is associated with the limbic circuitry. Apart from targeting the dorsomedial striatum and the nucleus accumbens output, the majority of cholinergic neurons in the LDT also project to the anterior pretectal nucleus, anterior intralaminar nucleus, anterior ventral thalamic nucleus, dorsomedial thalamic nucleus, central medial thalamic nucleus, and habenular nucleus.^10^ These nuclei, along with the PPN, play a role in modulating basal ganglia function and cholinergic transmission‐related neuropsychiatric disorders (e.g., PD, progressive supranuclear palsy).^56^, ^57^ As a primary member of the extrapyramidal system, the basal ganglion is a set of nuclei located in the deep ventral telencephalon, near the diencephalon and mesencephalon. It mainly consists of the striatum (caudate nucleus, putamen, and nucleus accumbens), inner/outer globus pallidus, reticular part of the substantia nigra, pars compacta of the substantia nigra, subthalamic nucleus, and hypothalamus. The striatum is the center of signal integration in the basal ganglia motor loop,^89^ receiving excitatory afferents from the cortex and thalamus, as well as the dopaminergic afferent from the midbrain, and transmitting to the downstream inner/outer globus pallidus and the reticular part of the substantia nigra. The output signals are further transmitted to the thalamus and affect the signal transduction of the basal ganglia motor loop via feedback.^90^ The striatum is divided into the following three parts along the dorsolateral to ventral medial axis: the dorsolateral caudate nucleus and putamen involved in the regulation of sensorimotor and cognition, and the middle part and the ventral nucleus accumbens and olfactory tubercle related to the regulation of marginal lobe function, such as cognition and emotion.^58^, ^59^

Cholinergic intermediate neurons account for only a small portion of the total cells in the striatum, with 1–3% in rodents and 20%^91^ in primates; however, they are the only neurons synthesizing ACh in the striatum.^92^, ^93^ The PPN and LDT contain a significant population of cholinergic neurons that regulate the activity of midbrain dopamine neurons by ACh signaling.^57^ They work together to maintain the ACh‐DA balance.^94^ Additionally, cholinergic neurons in the brainstem innervate the interlaminar and midline nuclei of the thalamus,^95^ thereby influencing the activity of the striatum. Abnormal function of cholinergic intermediate neurons may induce structural changes in striatal nuclei and disturbance of the local circuit,^96^ resulting in abnormalities of behavior and of other motor functions, ultimately leading to diseases such as PD,^40^ progressive supranuclear palsy,^60^ dystonia,^61^ and attention deficit hyperactivity disorder.^97^, ^98^ This indicates that the local cholinergic circuit of the striatum is involved in neurological diseases related to motor imbalance.

Recent studies^99^ based on positron emission tomography (PET) have found that cholinergic neurons in the PPN and LDT innervate the thalamus, including the lateral geniculate nucleus (LGN) and the medial geniculate nucleus (MGN). These findings suggest that the cholinergic projections may be involved in visual and auditory recognition impairments in patients with AD. The LGN serves as a relay station for visual information to the visual cortex and plays a facilitative role in coordinating cortical involvement in relevant visual stimuli and tasks.^100^ The MGN acts as a relay station in the auditory system and is involved in complex pitch processing and speech recognition, suggesting its involvement in cognitive activities.^101^ However, the underlying mechanisms of visual and auditory recognition impairments in patients with AD due to cholinergic deficits in the LGN and MGN still require further investigation.

## Summary and Prospect

The extensive and complex fiber projection of cholinergic neurons forms the cholinergic neural circuits, which regulate several nuclei in the brain through neurotransmission and participate in learning and memory, attention, emotion, movement, and so on. The loss of cholinergic neurotransmitters, the reduction, loss, and degeneration of cholinergic neurons or abnormal theta oscillations and cholinergic neural circuits can induce cognitive disorders such as AD, PD, PDD, and DLB. Further investigation of cholinergic fiber projections in various brain regions, such as MS, DBB, NBM, amygdala, and PPN, and further uncovering the underlying mechanisms of the neural circuits is critical to reveal the process of neurological diseases. However, the projection and function of cholinergic fibers in some nuclei of the brain, such as the HDB, SI, and medulla oblongata, remain unclear. The precise regulatory mechanisms of the cholinergic neural circuits in the brain also remain unclear. Furthermore, the diversity of postsynaptic cholinergic signaling pathways and heterogeneity of noncholinergic neurons has an unignorable influence on the study of neurological diseases. Therefore, to draw a more detailed topographic map of the central cholinergic nervous system, it is necessary to illuminate the projections of central cholinergic neurons and their modes of action. This will be helpful in the study of neural circuits and their regulation mechanisms for cognition, learning and memory, attention, sleep, fear, emotion, etc. However, with the aging of society becoming increasingly serious, PD, PDD, AD, and other senile cognitive disorders occur frequently. Further studies on the central cholinergic nervous system and its circuits are expected to open up new avenues for the prevention and treatment of senile neurodegenerative diseases.

## Author Contributions

GH outlined and wrote the manuscript. HZ, HD and YL provided guidance throughout the writing of the article. All the authors read and approved the final manuscript.

## Funding Information

This work was supported by the Beijing Natural Science Foundation of China (7222250).

## Conflict of Interest

The authors declare no conflict of interest.

## Data Availability

Not applicable.
